# Daily multidisciplinary rounds reduce ICU length of stay

**DOI:** 10.1186/cc10201

**Published:** 2011-06-22

**Authors:** ES Pacheco, IP Campos, JF Seixas, S Conejo, HP Vieira, SRG Mazutti, CFP Garcia, DT Noritomi

**Affiliations:** 1 Hospital Paulistano, São Paulo - SP, Brazil

## Introduction

Daily multidisciplinary rounds (DMR) can be helpful to improve communication, share common goals and result in better patient outcome [1].

## Objective

To evaluate the impact of the institution of DMR in clinical outcomes in a mixed ICU of a private hospital.

## Methods

DMR were instituted in our mixed tertiary 16-bed ICU in October 2010. Using our patient data bank (Epimed^©^) we retrieved admission clinical and demographic data and outcome information in two different admission periods: 1 year before and 1 year after institutions of DMR. Four independent multivariate analysis were performed with the ICU length of stay (LOS), hospital LOS, ICU mortality and hospital mortality as dependent variables. The independent variables were: period (previous to DMR and post DMR), age, SAPS III score, Charlson score and type of admission (clinical vs. scheduled surgery vs. unscheduled surgery).

## Results

From October 2008 to October 2010, 1,600 patients were admitted to our ICU: 656 in period 1 (before DMR) and 944 in period 2 (after DMR). There was no gender or age difference between the two periods. However, there were significant differences in the type of admission (more urgent surgery in period 1, *P *< 0.01), greater SAPS III (53.4 vs. 46. 4; *P *< 0.01) and Charlson score (2.9 vs. 1.7; *P *< 0.01) in period 1 in comparison with period 2. In the multivariate linear analysis, the ICU LOS was independently associated with the SAPS III (standardized beta = 0.17; *P *< 0.01) and period 2 - after DMR (standardized beta = -0.07; *P *= 0.01). Only the SAPS influenced hospital LOS (standardized beta = 0.27; *P *< 0.01). ICU mortality was only independently associated with SAPS III (standardized beta = 1.11; *P *< 0.01). Hospital mortality was independently associated with SAPS III (standardized beta = 1.09; *P *< 0.01) and Charlson score (standardized beta = 1.07; *P *= 0.02).

## Conclusion

The institution of multidisciplinary rounds was independently associated with a reduction in the ICU length of stay, without any significant effect in hospital outcomes.
